# The financial burden of out of pocket payments on medicines among households in Ethiopia: analysis of trends and contributing factors

**DOI:** 10.1186/s12889-023-15751-3

**Published:** 2023-05-03

**Authors:** Getahun Asmamaw Mekuria, Eskinder Eshetu Ali

**Affiliations:** 1 Department of Pharmacy, Arba Minch University, College of Medicine and Health Sciences, Arba Minch, Ethiopia; 2 Department of Pharmaceutics and Social Pharmacy, School of Pharmacy, College of Health Sciences, Addis Ababa University, Addis Ababa, Ethiopia

**Keywords:** Catastrophic medicine expenditure, Ethiopia, Financial risk protection, Out-of-pocket payments, Poverty, Universal health coverage

## Abstract

**Background:**

In Ethiopia, out-of-pocket (OOP) payment is the key means of healthcare financing, and expenses on medicines are a crucial component of such payment. This study aims to investigate the financial implications of OOP payments on medicines for Ethiopian households.

**Methods:**

The study involved a secondary data analysis of the national household consumption and expenditure surveys of 2010/11 and 2015/16. The "capacity-to-pay" method was used to calculate catastrophic OOP medicine expenditures. The extent of economic status related to catastrophic medicine payment inequity was calculated using concentration index estimation. The impoverishment consequences of OOP payment on medicine were estimated using poverty headcount and poverty gap analysis methods. Logistic regression models were used to identify the variables that predict catastrophic medicine payments.

**Results:**

Medicines accounted for the majority of healthcare spending (> 65%) across the surveys. From 2010 to 2016, the total percentage of households facing catastrophic medicine payments decreased from 1% to 0.73%. However, the actual number of people expected to have experienced catastrophic medicine payments increased from 399,174 to 401,519 people. Payment for medicines pushed 11,132 households into poverty in 2015/16. The majority of disparities were explained by economic status, place of residence, and type of health services.

**Conclusion:**

OOP payment on medicine accounted for the majority of total health expenses in Ethiopia. A high medicine OOP payment continued to push households into catastrophic payments and impoverishment. Household seeking inpatient care, those with lower economic status and urban residents were among the most affected. Hence, innovative approaches to improve the supply of medicines in public facilities especially those in urban settings and risk protection mechanisms for medicine expenditures particularly for inpatient care are recommended.

**Supplementary Information:**

The online version contains supplementary material available at 10.1186/s12889-023-15751-3.

## Introduction

In the context of healthcare, out of pocket (OOP) payment is the direct payment made by patients for the costs of healthcare services. OOP payment is regarded as a major hindrance factor for access to healthcare and cause for catastrophic health expenditure (CHE) [1]. According to the World Health Organization (WHO), CHE occurs when payment for healthcare services exceeds or equals 40% of non-food spending or 10% of total household consumption [2, 3]. Globally, up to 150 million people face CHE each year and up to 100 million are forced into poverty [3, 4]. The incidence of CHE is more common in lower-to-middle-income countries where up to 13.7% of households in some African countries face CHE at a 40% threshold of non-food expenditure, respectively [5–8].

OOP payments account for a major share of the Ethiopian health financing system. According to a report by the Ethiopian Ministry of Health (MOH), OOP health spending accounted for 31% of the total health spending in 2016/17 [9]. Studies conducted based on the Ethiopian household expenditure surveys reveal that the OOP health expenditures push close to a million people into poverty annually [10]. Ethiopia is now working on introducing financial protection measures, mostly through community based health insurance (CBHI) and a fee waiver system for the rural and vulnerable population [11, 12]. Even though the number of people enrolling in CBHI is growing, the proportion remained low [13]. As a result, a large proportion of Ethiopian households rely on OOP health expenditures [12].

The global trend shows that medicines remain to be the most expensive component of health services that account for the majority of OOP health payments [14–16]. In countries like India where over a billion people live, expenditure on medicines pushes close to 40 million people into poverty [17]. Despite the presence of a relatively better level of information in the rest of the world, there is limited evidence on the catastrophic and impoverishing effects of OOP medicine payments in Ethiopia. Previous studies in Ethiopia focused on the total catastrophic and impoverishing implications of OOP health expenditures in general [6, 10, 18]. As such, this study aimed to assess the catastrophic and impoverishing effects of OOP payments on medicines and its trend over time and the contributing factors in the Ethiopian context.

## Methods

### Data

This study analyzed secondary data from two consecutive nationally representative household consumption expenditure (HCE) surveys conducted in 2010/11 and 2015/16. The Ethiopian HCE surveys use cross-sectional study design to gather socioeconomic and demographic data of the households. The 2015/16 HCE survey included all rural and urban areas of the country, whereas the 2010/11 survey included all rural and urban areas of the country except pastoralist populations in Afar (three zones) and Somali (six zones) regional states [19, 20]. The survey emphasizes household expenditures, such as expenditures on food, non-food items, and OOP healthcare services. The expenses for healthcare services (including medicine) were gathered based on inpatient and outpatient services. The reference period was 30 days for outpatient services and one year for inpatient care. The detailed method used (such as study setting, study design, and data collection) by the Ethiopian central statistics agency (CSA) was presented in Additional file 1. The electronic version of the combined HCE dataset for both surveys was obtained in SPSS data format from the CSA.

This study used relevant survey data at the individual and household levels. Individual-level data included socioeconomic information (such as age, sex, education, and place of residence) as well as monthly healthcare service expenses. Household level data such as family size and total household consumption expenditures on food and non-food items were included in the analysis (Additional file 2).

### Outcome indicators

The financial burden of OOP payments on medicines was analyzed using four household level indicators. These are, (I) monthly OOP payment for medicines per person (adjusted for inflation), (II) monthly OOP payment for medicines as a share of household’s total and non-food expenses, (III) the number of households that faced catastrophic OOP medicines payments, and (IV) the number of households that fall under the poverty line after netting out medicine OOP payments from total household consumption expenses. Additionally, disparity distribution in healthcare financing was measured using concentration indices.

### Data analysis

All estimates in this study were sample weighted. Coded data were transferred to and analyzed using STATA 14.2, statistical software. In this study, the capacity-to-pay (CTP) approach was used. Consumption data were used because various studies, notably the WHO Xu model, have demonstrated that household consumption data could better reflect a household's economic position than household revenue (income) data, particularly in developing countries [1, 21]. According to WHO’s Xu model, the CTP of a household is defined as non-food expenditure that is computed by subtracting food expenditures from the total household expenditure. Thus,

$$CTP=Total\;household\;expenditure\;-\;Subsistence\;spending$$

However, if household food expenditure is less than subsistence spending CTP will be estimated using the following formula.

$$CTP=Total\;household\;expenditure\;-\;Food\;expenditure$$

Household subsistence spending is the bare minimum of expenses that a household must make to survive in the community, [1, 17, 22].

The poverty line (PL) was calculated based on household food consumption. According to Ethiopian HCE surveys household food consumption includes all the foodstuffs and the value of self-food consumption if it is produced at home. However, it excludes food feasting outdoors (hotel, restaurant…etc.), expenditures on tobacco and beverages such as alcohol [19, 20]. The PL is defined as a household's food consumption expenditures falling beneath the 50^th^ percentile of the country's food share expenditures [23]. To limit the estimation error, we calculated the national PL using the formula recommended by the WHO catastrophic expenditures methodological guideline [1]. As a result, the 45^th^ and 55^th^ percentiles of food consumption expenditure were considered. Furthermore, rather than using direct household size and food spending, equivalent household size and food expenditure were used. Equivalent household size is the number of consumption equivalents in the household estimated using the household scale parameter coefficient that is used to control the influence of factors among households. As a result, the equivalent household size was determined using the parameter coefficient of (ß = 0.56), which was reported in earlier studies conducted in 59 nations, [1, 22]. The food consumption was then adjusted for equivalent household size. This indicates that as the number of household members increases, the increase in consumption is less than proportional to the increase in household size. Furthermore, the international PL recommended by a world bank was utilized to compare Ethiopia's status with other countries, [24]. The worldwide PL cut point was set at USD 1.90 purchasing power parity for prices of the 2011–2012 period, [25]. The detailed methods utilized for estimation of PL, CTP, OOP and CHE due medicine OOP payment) can be seen in Annex II of Additional file 1.

To calculate household OOP payments, all types of direct payments to healthcare services were added together, including outpatient and inpatient transportation costs. Components to estimate OOP payment for outpatient care included medicine, X-ray, endoscopy, ultrasound, laboratory test (Excl. HIV), and other medical services, as well as doctor's visit and other medical services. Components considered for inpatient care were X-rays, endoscopy, ultrasound, laboratory tests (excluding HIV), doctor's visits, and other essential services, such as lodging (see Table A-1 in Additional file 2). The total household OOP payment on medicine during outpatient and inpatient care costs was determined by adjusting the spending to one month [1].

The magnitude of CHE was estimated using the method proposed by Wagstaff and Doorslaer (2003), [26]. When a household's OOP medicine payment for healthcare exceeds a predetermined threshold of the household's subsistence expenditure, the household incurs catastrophic medicine expenditure. The following formula was used to estimate the financial contributions to healthcare services and the headcount of CHE among the households.

$$CHE=\;(total\;household\;OOP\;payment/\;capacity\;to\;pay)>\;the\;threshold\;used\;to\;define\;CHE$$

A 40% cut-off point is preferred when utilizing the CTP strategy, while a 10% cut-off point technique is often used when using the household’s total income or expenditure, [2, 3]. However, several studies use arbitrary cut-off points of 5%, 10%, 25%, and 40% of CTP as suggested thresholds, [6–8]. In this study, a range of thresholds, such as 5%, 10%, 25%, and 40%, were utilized to investigate the impact of outcomes and to compare study findings to those of other similar investigations.

Overshoot (intensity) was also estimated to capture the extent to which individual health/medicine payments surpassed the stated threshold for households that incurred catastrophic expenditures. It is essential because the CHE headcount is unable to record the extent of OOP payment once a particular threshold has been exceeded (see Annex II of Additional file 1).

The PL and the proportion of total food expenditure were used to determine poverty. A household is considered impoverished if one falls below the estimated PL, after paying for health services (or medicine) (see Annex II of Additional file 1).

### Poverty headcount and poverty gap

In this study, the impoverishment effect of OOP medicine payment was calculated using the estimated PL as follows:

$$PL=\;(\sum\;(WGTh\;\ast\;Equ\_foodexph)\;)/(\sum WGTh);\;Where\;45^{th}<\;Food\_exph\;<55^{th}$$

*Where*:- PL: Poverty Line and WGT_h_: Weight of households

The difference between the gross poverty headcount (before medicine payments) and the poverty headcount (after medicine payments) was calculated [1]. Deducting household per capita expenditure from the PL yields the gap between the poor and the PL. This step only includes poor households. As a result, the magnitude of poverty can be determined based on the living standard [1].

#### Estimating predictors 

Logistic regression models were used to estimate the effects of independent variables (socioeconomic characteristics and types of healthcare services) on the likelihood of incurring catastrophic medicine expenditure. Bivariate and simultaneous logistic regression models were applied sequentially. First, a bivariate regression model was performed, and variables with *P* ≤ 0.25 were included in a simultaneous logistic regression. In this study, the logistic model used household per-capita expenditure as a continuous variable. Hence, the chance of facing catastrophe rising in each fraction of per-person medicine expenditure was estimated. This could be an indicator of the socioeconomic perspective. A statistically significant association was revealed once the *P*-value at the 95% confidence interval (CI) was < 0.05. The adjusted odds ratio (AOR) was also calculated for covariates selected for simultaneous logistic regression. Multicollinearity test was also conducted among dependent and independent variables and no harmful collinearity was detected in the model (mean Variance Inflation Factors (VIF) = 1.23 in 2015/16 and 1.21 in 2010/11 survey years).

## Results 

### Demographic and socio-economic profile of households

This section summarizes the results of household surveys conducted in the 2010/11 and 2015/16 survey years (Table 1). Non-zero health spending was reported by 15,961 households in 2010/11 and 18,585 households in 2015/16. In 2010/11 and 2015/16, 9,531 (59.7%) and 12,426 (66.9%) of urban residents reported spending on healthcare services, respectively. In the two surveys, the average household size was 6 (SD = 2.5) and 5 (SD = 2.3), respectively. In 2010/11 and 2015/16, the average age of households was 22.6 (SD = 17.9) and 23.3 (SD = 17.9), respectively. In 2010/11, 10,078 (63.2%) households had individuals with a lower level of education (elementary), whereas only 276 (1.3%) households had members with a tertiary level of education. Household members with primary school education or less and those with a higher educational level respectively made up 5,971 (32.1%) and 7,004 (37.7%) of the surveyed population in 2015/16. The majority of households in both surveys were headed by men. In 2010/11 and 2015/16, only 6 (0.04%) and 17 (0.09%) households had health insurance, respectively. OOP health payments for inpatient and outpatient services were respectively reported by 14,595 (78.5%) and 6,344 (34.1%) households in 2015/16.

**Table 1:** Socio-demographic profile of households who reported non-zero health expenditure in 2010/11 and 2015/16

**Variable**	**Year**	
	**2010/11**	**2015/16**
	**n (%)**	**n (%)**
Household head gender		
Male	10,937 (68.54)	11,373 (61.2)
Female	5,024 (31.5)	7,212 (38.8)
Location		
Rural	6,430 (40.3)	6,159 (33.14)
Urban	9,531 (59.7)	12,426 (66.9)
Household size (equivalent) (Eq. 2 in Additional file 1)		
1	4,309 (27)	4,255 (22.9)
2	2,545 (16)	9,128 (49)
3	2,517 (15.8)	4,762 (26)
4	3,534 (22.1)	419 (2.3)
5 +	3,056 (19.13)	21 (0.13)
Household age composition		
≤ 18	1,173 (11)	310 (1.7)
19–35	7,310 (45.8)	6,707 (36.1)
36–54	3,254 (20.4)	7,460 (40.1)
> 55	3,623 (22.7)	4,108 (22.1)
Education of household head		
None	4,985 (31.2)	3,794 (20.4)
Primary	10,078 (63.2)	5,971 (32.1)
Secondary	622 (3.9)	1,816 (9.8)
Post-secondary	276 (1.3)	7,004 (37.7)
Employment of household head		
Employed	1,839 (11.5)	15, 775 (84.9)
Unemployed	14,122 (88.5)	2,810 (15.1)
Type of health service		
In-patient service	NA	14,595 (78.5)
Out-patient service	NA	6,344 (34.1)
Insurance		
Insured	6 (0.04)	17 (0.09)

*NA* Information not available

### Monthly household medicine expenditures

As shown in Table 2, in 2010/11, the average monthly household expenditure was 1,219.7 ETB (SD = 1,555) (29.9 USD) and in 2015/16, it was 2,841.4 ETB (SD = 4,150.5) (69.6 USD). In the meantime, food accounted for around 46% (SD = 14) of household expenditure in 2010/11 and 51% (SD = 15.6) in 2015/16. The percentage of households obtaining medicine through OOP payment increased from 88% in 2010/11 to 95% in 2015/16. OOP payments on medicine account for nearly 66% of total healthcare services across both surveys.

**Table 2:** Financial burden indicators, Ethiopia, 2011/2012 and 2016/16

Variables	Types of expenditures	2010/11	2015/16	t-test	*P*-value
		Mean (SD,)	Mean (SD,)		
Monthly per capita expenses (ETB)^b^	HHs expenditure	1,219.7 (1,555)	2,841.4 (4,150.5)	-48.6^a^	0.000
	Health^c^	32.7 (93)	52.6 (189.2)	-12.1^a^	0.000
	Medicine^c^	12.2 (54.5)	13.9 (80.1)	-2.35^a^	0.018
Share of OOP payment on medicine	HHs OOP expenditure	88.5% (6.3%)	95.2% (2.2%)	-0.0014^a^	0.000
Share of total HH expenditure (%)	Health^c^	3.5% (10.8%)	3% (18.8%)	1.12^a^	0.27
	Medicine^c^	1.3% (5.08%)	0.8% (4%)	10.6^a^	0.000
Share of non-food HH expenditure (%)	Health^c^	6.7% (25.8%)	8.4% (11.4%)	-0.93^a^	0.354
	Medicine^c^	2.6% (13.9%)	4.8% (10.0%)	6.35^a^	0.000

Figures in brackets are standard deviations (SD)

OOP out of Pocket Payment, HH Household

^a^independent samples t-test

^b^ETB at constant 2009–2010 prices

^c^OOP expenditure on health and medicine-only

The average OOP payment for healthcare services has risen by 38%, from 32.7 ETB (0.75%) in 2010/11 to 52.6 ETB (1.3 USD) in 2015/16 (*P* = 0.000). During the same period, the average cost of medicine increased by 12% (*P* = 0.018). However, the proportion of OOP payment on medicine as a share of total household expenditure was reduced in the most recent survey. Interestingly, the proportion of OOP payment on medicine as a share of CTP (non-food expenditure) grew by about 2%. Medicine OOP spending increased significantly over the two survey years.

#### Monthly expenditure by quintile

In 2010/11, the richest quintile spent roughly 4 (*P* = 0.001) times more on medicines than the lowest quintile, and 3 (*P* = 0.000) times more in 2015/16 (see Table A-3 in Additional file 2). However, the gap between the second, middle, and fourth average expenditure quintiles of both surveys were narrower (< 5%).

#### Incidence, intensity, and distribution of catastrophic medicine expenditures

A percentage of OOP medicine payments to subsistence expenses has been calculated using a set of thresholds (Table 3). In 2010/11, when the cut-off levels were increased from 5 to 40% of non-food household expenditure, 10.9% (at the 5% threshold) and 1% (at the 40% threshold) of households experienced CHE as a result of medicine OOP payment. In 2015/16, the percentage of households that incurred CHE were 7.1% and 0.73% at thresholds 5% and 40% of non-food expenditure, respectively. In 2015/16 and 2010/11, a total of 399,174 and 401,519 people were affected by catastrophic medicine payments at a 40% threshold (see Table A-5 in Additional file 2).

**Table 3:** Incidence, intensity and distribution of catastrophic health and medicine payment among households in Ethiopia, 2010/2011 & 2015/2016

**Year**		**2010/2011**				**2015/2016**			
**Threshold level (Z**_**cat)**_		***5%***	***10%***	***25%***	***40%***	***5%***	***10%***	***25%***	***40%***
Variables	Type of expenditure	**Headcount measures**							
H_cat_	Health	26.69%	14.56%	5.28%	2.70%	24.65%	14.12%	5.37%	3.18%
	Medicine	10.90%	5.87%	1.87%	1%	7.10%	4%	1.40%	0.73%
C^E^	Health	0.101	0.1247	0.1516	0.1754	-0.1771	-0.2175	-0.2852	-0.3013
	Medicine	0.1272	0.1498	0.000	0.2627	-0.1482	-0.179	0.000	-0.3737
H^**w**^	Health	24.00%	12.70%	4.50%	2.20%	29.00%	17.20%	6.90%	3.10%
	Medicine	9.50%	5.00%	1.90%	0.70%	8.10%	4.80%	1.40%	1.00%
**Gap measures**									
G_cat_	Health	4.35%	3.36%	2.06%	1.50%	8.43%	8.42%	8.41%	8.40%
	Medicine	0.35%	3%	10.70%	18.30%	1.10%	0.86%	0.50%	0.40%
C^O^	Health	0.186	0.2066	0.2427	0.2761	0.364	0.365	0.365	0.366
	Medicine	-0.5243	-0.2124	0.000	0.000	-0.3046	-0.3479	0.000	0.000
O^W^	Health	4%	3%	2%	1%	5.36%	5.35%	5.33%	1.33%
	Medicine	1%	4%	1%	1%	1.50%	1.20%	0.50%	0.40%
MPG_cat_	Health	16.67%	23.62%	44.44%	45.45%	18.48%	31.10%	77.25%	42.90%
	Medicine	11%	80%	58%	71%	19%	25%	36%	40%

*Z*_*cat*_ set of cut-offs, *H*_*cat*_ catastrophe headcount, *C*^*E*^ concentration expenditure indices, *H*^*w*^ weighted headcount

*G*_*cat*_ catastrophe overshoot, *C*^*O*^ concentration expenditure, *O*^*W*^ weighted overshoot, *MPG*_*cat*_ Mean positive gap

Across all thresholds (5% to 40%), the mean overshoot (intensity) of medicine catastrophic payment was lower in 2015/16 than in 2010/11. For example, between 2010/11 and 2015/16, the overshoot dropped from 18.3% to 0.4% at the 40% cut-off point. Unlike incidence and intensity, mean overshoot crosses the thresholds in the opposite direction. In 2015/16, for example, households facing CHE at a 5% threshold spent an average of 24% (19% + 5%) of their monthly non-food expenditure on medicines. However, the average proportion increased to 80% (40% + 40%) at a 40% threshold.

The incidence concentration indices were positive in 2010/11 but negative in 2015/16 across all sets of thresholds. The weighted CHE headcount score was lower than the unweighted CHE headcount score in 2010/11. However, the weighted CHE headcount scored higher in 2015/16. The weighted overshoot exceeded the unweighted overshoot in both survey years. Both surveys had negative overshoot indices attributed to OOP payment on medicines.

#### Catastrophic medicine payment by quintile

**Fig. 1:**
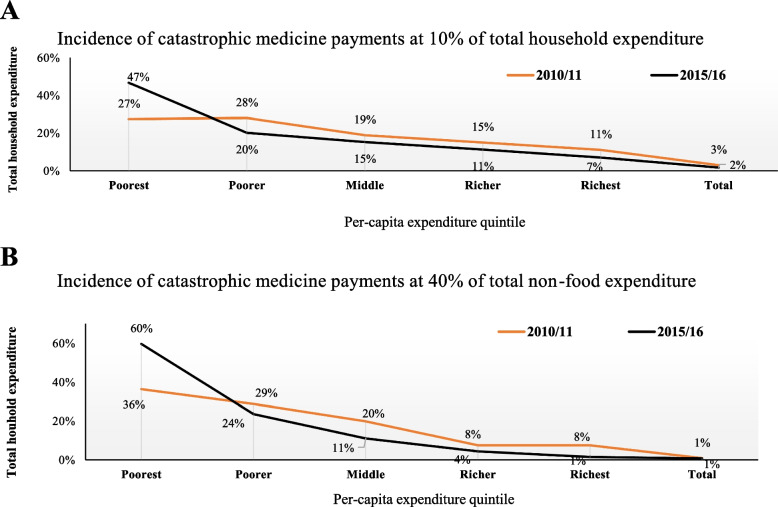
Households experiencing catastrophic payments owing to medicine OOP payment by quintile at 10% and 40% of total expenditure

When comparing the poorest quintiles to the other quintiles, the trend from 2010/11 to 2015/16 demonstrates that the poorest quintiles are experiencing CHE (Fig. 1). However, across surveys, the incidence of CHE is marginally decreasing for the remaining quintiles.

#### Medicine expenditures and poverty

Table 4 gives a poverty headcount and gap estimate before and after netting out medicine payments in Ethiopia to examine the sensitivity of poverty to healthcare services. In this study, the estimated poverty level per capita per month was 392.94 ETB (USD 17.7) in 2010/11 and 872.04 ETB (USD 50.8) in 2015/16 (based on survey data).

**Table 4:** Impoverishment impact of OOP payment attributed to medicine expense among Ethiopian households in 2010/11 & 2015/16

	**Gross health payment (1)**^**b**^		**Net of health payment (2)**^**c**^		**Difference**			
					**Absolute** **(3) = (2)-(1)**		**Relative** [(3)/ (1)] *100	
**Year**	**2010/11**	**2015/16**	**2010/11**	**2015/16**	**2010/11**	**2015/16**	**2010/11**	**2015/16**
Poverty line (ETB/Month)	392.94	872.04	392.94	872.04	392.94	872.04	392.94	872.04
Poverty headcount								
Health	0.63%	1.5%	1%	1.63%	0.4%	0.1%	6.3%	9%
Medicine			0.8%	1.52%	0.17%	0.02%	1.7%	1.3%
Poverty Gap (ETB current price ^a^)								
Health	64.7	130.1	103.9	180.2	39.2	50.1	60.6%	38.5%
Medicine			111.4	184.6	46.7	54.5	72.2%	41.9%
Normalized poverty gaps								
Health	3.5%	8.8%	14.7%	12.1%	11.2%	3.4%	3.2%	38.5%
Medicine			15.8%	12.44%	12.3%	3.7%	3.52%	41.9%

^a^current price was calculated using the Consumer Price Index (CPI) ratio converting method

^b^The poverty headcount and gap estimate before netting out the health & medicine expenditure

^c^The poverty headcount and gap estimate after netting out the health & medicine expenditure

According to the estimated PL, about 0.63% and 1.5% of those who reported non-zero OOP payments for health were under the PL in 2010/11 and 2015/16, respectively. When the OOP payment on medicines is netted out, the share of poor households rises to 2.7% and 1.52% in the respective surveys. Medicine OOP payments pushed 74,144 people into poverty in 2010/11 and 11,132 people into poverty in 2015/16 (Table A-5 in Additional file 2). OOP medicine expenses increased the poverty estimation by 1.7% and 1.3% in the 2010/11 and 2015/16 survey years, respectively. OOP total health spending increased poverty rates by 6.3% (in 2010/11) and 9% (in 2015/16). Based on the international PL of USD 1.90 per day, OOP payments on medicine increased the poverty estimation by 5.1% and 12% in 2010/11 and 2015/16, respectively (Table A-4 in Additional file 2).

Between 2010/11 and 2015/16, the gross poverty gap increased from 64.7 ETB (USD 1.6) to 103.9 ETB (USD 2.5). During the same period, the poverty gap attributed to OOP medicine payments increased from 46.7 ETB (USD 1.1) to 54.5 ETB (USD 1.3). The OOP payments on medicines increased the absolute normalized poverty gap (as a percentage of the PL) by 12.3% (in 2010/11) and 3.7% (in 2015/16).

The trend of our estimates suggested that OOP payments on medicines have continued to play a significant role in the impoverishment impact of total health expenditure. Thus, OOP payments for medicines represent a higher share of total health spending (Table 3).

#### Impoverishment by place of residence and expenditure quintile

**Fig. 2:**
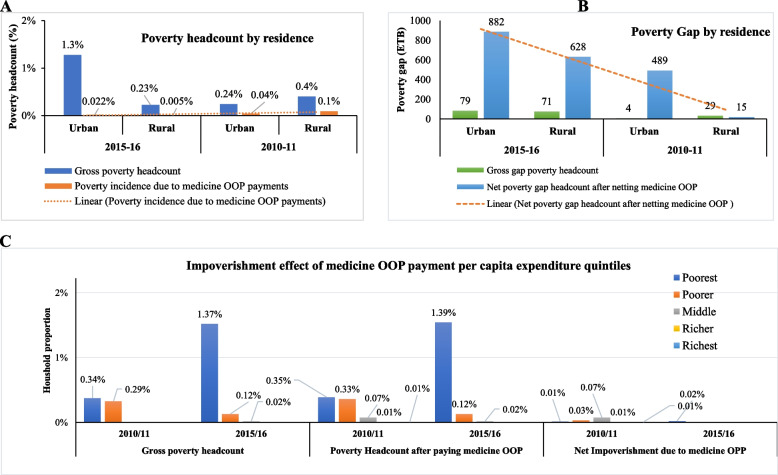
Impoverishment effect of medicine OOP payment by residence and per capita expenditure quintile among households in Ethiopia during 2010/11 and 2015/16

In both surveys, OOP payment for medicines increased poverty headcount more in urban areas than in rural areas (Fig. 2 (A and B). In 2015/16, medicine OOP payments increased poverty estimates in urban and rural areas by 10.2% and 7.8%, respectively, compared to 13.6% and 6.4% in 2010/11. The first to middle quintiles of expenditure was mainly vulnerable to impoverishment due to OOP payments on medicines across the survey years (Fig. 2 (C)). Compared to the fourth and fifth quintiles of the two surveys, the proportion of impoverished households owing to medicine expenditure was lower in the 2015/16 survey year.

#### Factors associated with catastrophic medicine expenditure

Bivariate analysis identified five variables (in 2010/11) and seven (in 2015/16) to be significant. Across both surveys, the status of health insurance, place of residence, and expenditure quintile were associated with catastrophic medicine expenditure (Table A-5 in Additional file 2). Seeking inpatient care was an additional significant factor in incurring catastrophic medicine expenses in 2015/16. However, there was some variation in the likelihood of facing catastrophic medicine expenditure and household characteristics (such as gender, level of education, and employment status) over the survey years.

As shown in Table 5, in the simultaneous regression, four covariates (place of residence, household size, expenditure quintile, and per-capita medicine expense) were significantly associated with the dependent variable (facing catastrophic medicine expenditure) across surveys. Interestingly, the status of health insurance was statistically significant in 2010/11 but its significance was lost in 2015/16. Additionally, seeking inpatient care was another significant variable in 2015/16. The strongest variable that predicted facing catastrophic medicine expenditure in 2010/11 was the place of residence. The odds of rural residents facing catastrophic medicine expenditure was 1.84 (*P* < 0.001) times higher than urban residents. However, in 2015/16, urban residents were 60.8% more likely to face catastrophic medicine expenditure than rural residents (AOR: 0.392; *P* < 0.001). In 2015/16, the strongest variable that predicted facing catastrophic medicine expenditure was households having members aged ≥ 55 years old. The odds of households with elderly members facing catastrophic medicine expenditure were 1.3 (*P* = 0.217) times higher than households having members aged ≤ 55 years old. Inpatient health services increase the risk of catastrophic medicine expenditure by 58.3% more than outpatient care in 2015/16 (AOR: 0.417; *P* < 0.001).

**Table 5:** Simultaneous logistic regression of factors associated with household catastrophic medicine expenditure at OOP/CTP ≥ 0.40; Ethiopian HCE survey 2010/11 and 2015/16

**2010/11**						
**Covariates**	**S. E**	**Wald Chi**^**2**^	**Sig**	**AOR**	**95% CI**	
Rural residence	0.033	287.4	0.000	1.842	1.464	2.317
≥ 5 members living in HH	0.006	287.7	0.028	1.057	0.999	1.119
≥ 55 years old member living in HH	0.010	285.5	0.811	1.101	0.999	1.214
Unemployed HH head	0.068	296.7	0.950	0.017	0.002	0.121
Richest household	0.099	48.6	0.000	0.704	0.645	0.768
Lack of health insurance	0.117	6.74	0.000	1.008	-3.026	-2.56
High per-capita medicine expense^a^	0.0004	7.82	0.000	0.999	0.998	1.000
**2015/16**						
Female as a HH head	0.015	10.62	0.836	1.157	0.972	1.377
Rural residence	0.016	63.04	0.000	0.392	0.313	0.489
≥ 5 members living in HH	0.003	34.76	0.000	0.898	0.858	0.939
≥ 55 old members living in HH	0.020	17.85	0.217	1.308	1.049	1.631
HH head illiterate/primary	0.006	16.23	0.902	0.918	0.851	0.990
Outpatient service	0.018	28.12	0.000	0.417	0.317	0.549
Richest household	0.001	253.06	0.000	0.387	0.352	0.426
Lack of health insurance	0.016	3.41	0.083	1.04	-0.002	0.035
High per-capita medicine expense ^a^	0.007	343.09	0.000	1.014	0.0065	0.007

^a^Per capita medicine expenditure of household taken as a continuous variable; *S.E* Standard Error, *Sig* Significance, *AOR* Adjusted Odds Ratio, *CI* Confidence Interval

## Discussion

The present study illustrated that the prevalence of OOP payments on medicine has grown over the survey years of 2010/11 and 2015/16. Three popular techniques for calculating CHE headcount among households are CTP, household’s total income, and consumption. In the present study, the CTP was used as the denominator to calculate the cost of catastrophic medicine expenditure. Consumption data were used because various studies, notably the WHO Xu model, have demonstrated that household consumption data could better reflect a household's economic position than household revenue (income) data, particularly in developing countries [1, 21].

An overall 88% of people paid for medicines OOP in 2010/11 and this rose to nearly 95% in 2015/16. This figure was even higher than some African countries' OOP health expenditure (including medicine), such as Cameroon (66%) and Nigeria (90%), [27]. Moreover, the rate of OOP payment on medicine accounted for 79% in India, [17, 27]. These findings established the fact that OOP payments are the primary methods of financing medicine needs among Ethiopian households [4, 28, 29].

In the present study, 399,174 in 2010/11, and 401,519 in 2015/16 people had CHE due to OOP medicine expenses (at a 40% threshold). Despite the lack of reports on the impact of OOP medicine payment in Ethiopia and other African countries, high OOP health expenditure persists despite financial risk protection strategies. A high rate of OOP health expenditure and CHE headcount was also reported in Kenya, Cameroon, and Nigeria after ten years of implementation of health insurance [7, 8, 27]. Likewise, in Ethiopia, according to a review by Borde et al. (2022), the pooled CHE due to OOP expenses during healthcare services was 40.1% at the 10% threshold of non-food expenditure [18].

In the current study, there was a disparity in the distribution of household medicine spending and CHE head counts. For example, there was a significant (*P* = 0.018) disparity in per-capita medicine expenditure among the quintiles over the two survey years. In both surveys, the richest quintile had significantly higher medicine expenses, whereas poor households incurred the majority of the financial burdens. This implies that the impact of OOP payments on medicine has shifted from better-off to poorer households [1, 30]. This finding was consistent with a study from India [17].

Furthermore, OOP payments on medicine pushed 74,144 (0.17%) and 11,132 (0.02%) people into poverty in 2010/11 and 2015/16, respectively. Thus, even though 0.17% and 0.02% of people were not considered poor, they may be considered poor if the expenses of medicine are deducted [26, 31]. Furthermore, between 2010/11 and 2015/16, the poverty gap due to medicine OOP payment increased from 46.7 ETB (USD 1.1) to 54.5 ETB (USD 1.3). In the same period, the normalized poverty gap (as a percentage of the poverty line) grew by 12.3% and 3.7%, respectively. Our estimate suggests that the widening of both gaps over the survey years indicates that more people have been forced to live in poverty due to OOP payments on medicines [26, 31]. The consequence of the high prevalence of poverty was associated with difficulties in access to healthcare in many developing countries [21]. In this study, the food share of total monthly household expenditure increased from 46 to 50% from 2010/11 to 2015/16, respectively. A higher proportion of poverty among households can be expected since food expenditure takes almost half of the households’ expenditure [22]. In this study, total household expenditure has risen in the most recent survey, which explains the lesser proportion of households’ expenditure on medicines. These findings can suggest that households might be spending on food and health to survive by minimizing their subsistence expenditures. A study on consumer behavior illustrated that the increased share of food expenditure means, households were prioritizing expenses for food [21]. Additionally, healthcare expenses are obligatory as compared to other non-food expenditures [32].

In terms of associated factors, OOP payment for inpatient care increased the likelihood of CHE by 64% (*P* < 0.001) in 2015/16. This figure is consistent with the findings of a Myanmar study, which stated that households admitted to health facilities were 7.8 times more likely to incur CHE than those who received outpatient care [33]. In this study, the likelihood of poor households incurring CHE was 30.6% in 2010/11 and increased to 65.8% in 2015/16. This finding is consistent with previous Ethiopian studies [12, 18]. The place of residence was another significant variable. Our estimates showed that the risk of incurring CHE due to OOP payment on medicine has shifted from rural to urban residents in the respective survey years. The findings of this study could indicate that households are obtaining medicines from private drug outlets which may lead to higher drug prices in urban areas.

Our estimates demonstrated the persistence of high OOP payments on medicine, their importance in dealing with CHE, the impact on impoverishment, and the worse-off poorer, inpatient care and urban residents of Ethiopian households. The possible explanation for the persistence might be associated with the limitations of financial protection strategies in improving access to medicines. Financial protection schemes are designed to minimize risk across vulnerable populations [34]. Ethiopia implemented CBHI and fee waiver schemes in 2010/11 [35]. However, according to the findings of this study, having health insurance no longer seems to be able to counteract the influence of other CHE variables (such as socioeconomic characteristics). Another possible explanation might be a lack of access to essential medicines in public health facilities. Ensuring the accessibility of medicine is a remarkable work to bring equitable health access, [36, 37]. Medicine accessibility is dependent on the availability and affordability of medicines, [38]. However, our findings point to Ethiopia's current situation in which the majority of commonly prescribed medicines in the country are costly and unaffordable [28, 39, 40]. Unfortunately, freely provided medicines by the public sector are often unavailable in in the facilities [28, 40]. Consequently, unavailability may lead to households turning to private healthcare facilities, potentially resulting in catastrophic medicine costs [41]. Unavailability and unaffordability are significant barriers to household access to medicine, particularly for poorer [14]. Hence, if households are required to pay for medicines, it may account for the majority of their non-food expenditure [26, 30].

In general, the findings of this study could lead to a better understanding of the burden of medicine OOP payments on access to healthcare. The persistence of financial hardship may be a considered as an indicator of the low level of effectiveness of various financial risk policies implemented in the country. The effectiveness of financial protective measures such as health insurance, fee waivers, and drug supply systems should be further investigated in Ethiopia. Further studies are also required to evaluate the burden of OOP payment on medicine disaggregated by region, as it is important to show regional variation and policy implications.

### Study limitations and strengths 

The present study provided evidence on the trends of financial implications of OOP payments on medicines and contributing factors in Ethiopia. Despite using a very large dataset and documenting trends over time, this study has some potential limitations. The following are limitations due to the method used in the data collection of the surveys. First, self-reported expenditures and recalling bias (particularly inpatient care charges requiring a 365-day memory) could be a source of bias. Second, data from households that did not seek healthcare due to financial constraints were also excluded from the surveys. Because this is a cross-sectional study, it does not consider the potentially catastrophic consequences of illness or sequelae (such as lost wages). The types of medicines used in this study were not specified. Finally, the financial implication of OOP payment on medicines was not disaggregated into regions.

However, the presence of limitations in the study cannot invalidate the findings. Because all acceptable standards were applied strictly to measure the catastrophic and impoverishing effects of OOP payments on medicines. As a result, this study contributes to identifying which types of healthcare services are largely responsible for catastrophic health spending and impoverishment.

## Conclusion 

Our estimates showed the persistence of high OOP payments on medicine across the survey years. Over the study periods, medicine payment covers the majority of healthcare expenses (> 65%). A high medicine OOP payment continued to push households into catastrophic payments, which resulted in impoverishment, resulting in a considerable increase in the country's estimated poverty level. During the study periods, the financial burden was worse-off in inpatient care-seeking households, poor households, and urban residents. Furthermore, the trend indicates that having health insurance is insufficient to financially protect enough households.

### Policy implications

The findings of our study have policy implications for decision-makers in Ethiopia and comparable countries. Since there was a high OOP medicine expenditure, that suggests people (particularly the poor) were having difficulty accessing medicines. Hence, minimizing the financial burden due to medicine expenditure can make a difference in our context. Therefore, the government, policymakers, and other concerned bodies should give attention to medicine-specific policies. To increase the effectiveness of the financial protection schemes, increasing the provision of medicines through health insurance schemes, improving free (for poor households) or low-cost medicine availability, and expanding their coverage to all communities of the country are required. Second, the medicine supply chain management system should be improved to enhance the availability of low-cost generic essential medicines at public health facilities. Finally, the findings of this study serve as a guide for future health financing reforms and a baseline for future studies on financial risk protection strategies in Ethiopia.

## Supplementary Information

**Additional file 1. **

**Additional file 2. **

## Data Availability

The data that support the findings of this study are available from the Ethiopian Central Statistical Agency but restrictions apply to the availability of these data, which were used under license for the current study, and so are not publicly available. Data are however available from the corresponding author upon reasonable request and with permission of the Ethiopian Central Statistical Agency. The Ethiopian Central Statistical Agency can also be directly contacted for the dataset used in this study.

## Abbreviations

AAU: Addis Ababa University
CBHI: Community Based Health Insurance
CHE: Catastrophic Health Expenditure
CSA: Central Statistical Agency
EHIA: Ethiopian Health Insurance Agency
ETB: Ethiopian Birr
FMoH: Federal Ministry of Health
HCE: Household Consumption and Expenditure
HH: Household
IMF: International Monetary Fund
MoF: Ministry of Finance
OOP: Out of Pocket
PPP: Per Person Payment
SES: Socio-Economic Status
SHI: Social Health Insurance
USD: United States Dollar
VIF: Variance Inflation Factors
WHO: World Health Organization
