# Supplementary material for: The financial burden of out of pocket payments on medicines among households in Ethiopia: analysis of trends and contributing factors
Source: BMC Public Health. 2023 May 3;23:808. doi: 10.1186/s12889-023-15751-3 (PMC10155387; doi:10.1186/s12889-023-15751-3)
Supplement: Supplementary file 1 — Additional file 1. [file 12889_2023_15751_MOESM1_ESM.pdf]

Annexure

Annex I: Method utilized by Ethiopian CSA for Household Consumption Expenditure (HCE) data collection

Ethiopia borders Kenya, Sudan, Eritrea, Djibouti, and Somalia and is a landlocked country in East Africa. The country is divided into ten regional states and two municipal administrations. Ethiopia's population is expected to exceed 108 million by July 2020 [1]. In 2010/11 and2015/16, the Ethiopian Central Statistics Agency (CSA) conducted countrywide household consumption and spending surveys [2]. Using a multistage sampling design, the country was divided into four categories such as rural, large urban-rural, major urban centers, medium towns, and small towns. This was required in order for the sample to be representative. For example, the rural and major urban-rural categories used a two-stage cluster sampling method, with primary sample units being Enumeration Areas (EAs) and secondary sampling units being twelve and sixteen homes, respectively. To town categories, it employed a three-stage sampling strategy that included towns as a primary sampling unit. The survey year's data gathering period encompassed every month of the year. This may allow it to deal with seasonality. The data collection activities in a single EA take fifteen days. During the survey week, data was collected twice from each sample household. A side-by-side survey of a nearby marketplace is conducted to compensate for the missing quantity pricing data. In addition, each round of surveys introduces new technologies to make data collecting easier, such as digital data collection in the 2015/16 survey. Using Probability Proportional to Size (PPS) methodologies, the sample size (number of households) was determined using national census data. In 2010/11 and 2015/16, a total of 27,834 and 30,229 households were polled, respectively [2]. In 2010/11, the number of household was estimated to be 15,975,055 (Sample Estimation (S.E)=155,400) and the population was estimated to be 76,100,054

(S.E=873,638) [3]. In 2015/16, the population and number of households were estimated to be 89,453,119 (S.E=896,884) and 19,399,161 (S.E=166,503), respectively [4].

Annex II: Estimation of financial burden indicators of out-of-pocket medicine expenditure

A. Estimating the household subsistence spending and poverty line

Based on the equivalent family size, the corresponding household food consumption was calculated. Furthermore, the weight of households was taken into account throughout the procedures. Therefore,

The equivalent food share expenditure was computed as:

$$Food\_exph = \frac{(Food_{Hh})}{Xi} \text{---} (1)$$

$$Equ_{\_sizeh} = H\_size_h^{\beta} \text{---} (2), \text{ then}$$

$$Equ\_food_{exph} = \frac{Food\_Hh}{Equ\_sizeh} \text{---} (3)$$

Where:

$Equ_{\_sizeh}$	=	Equivalent household size
Equ_food_{exph}	=	Equivalent food expenditure of household
$Food_{\_exph}$	=	Food share expenditure of household
$Food_{\_Hh}$	=	Total food expenditure of household
$H_{\_sizeh}$	=	Household size
Xi	=	Total household expenditure
β	=	Scale multiplier coefficient of household

The following formula shows how the PL was calculated.

$$PL = \frac{\sum (WGTh * Equ\_foodexph)}{\sum WGTh} \quad \text{Where } 45^{th} < Food\_exp_h < 55^{th} \text{ ----------- } (4)$$

Where: - PL: Poverty Line and WGT_h: Weight of households

The subsistence expenditure was calculated as:

$Subc_{exp} = PL * Equ\_size_h$ --(5)

Where $Subc_{exp}$ is the subsistence spending of household. The CTP of household was then estimated as:

$ctp_H = Xi - \text{Subcexp}$ --(6)

(If household food expenditures are less than subsistence spending); otherwise,

$ctp_H = Xi - Food_{Hh}$--(7)

where ctp_H is the ability-to-pay of households.

B. Measuring out of pocket payment

Constant prices from 2009-2010 were used to convert to current prices (March of 2021). It has been computed using values of the consumer price indices (CPI) ratio. (see Table A-2 in Additional file 2) The following formula was used to convert to current constant prices:

$$Current\ price = \frac{New\ CPI}{Old\ CPI} * (old\ price) \text{--(8)}$$

Where:

Old CPI = Health consumer price index of 2010/11 and 2015/16

New CPI = Health consumer price index of 2021

Old price = The nominal local price of health expenditures in 2010/11 and 2015/16

C. Per person monthly OOP payment and share of medicine OOP payment

The monthly OOP payment per person (PPP) is a ratio of the total monthly OOP payment to the size of the family. The ratio of total health/medicine OOP (Ti) to total household spending (Xi) or total non-food (nf(x)) expenditure was used to calculate the share of health/medicine costs (Sh_{OOP}).

As a result, the variables (total health spending and medicine-only expenditure) were employed interchangeably in the following calculations as needed in this investigation. Therefore,

$Sh_{OOP} = T_i / nf(x)$ --- (9)

Where:

Sh_{OOP} = Share of OOP payment (total health/medicine-only)

T_i= Total household health/medicine OOP

nf(x) = Total household non-food expenditure

D. Measuring the incidence of catastrophic medicine expenditure

The total household expenditure (Xi) is also used as a denominator to determine the proportion of household OOP expenditure on medicine. In addition, the impact of a household's total health spending was calculated to compare medicine-only expenditure. The number of households facing catastrophic expenditure was computed as follows:

$$\frac{Ti}{nf(x)} > Zcat \text{ ---(10)}$$

$$Ei=1 \text{ if } \frac{Ti}{nf(x)} \geq Zcat, \text{ and } Ei=0 \text{ when, } \frac{Ti}{nf(x)} < Z\,cat \text{ ---------------------------(11)}$$

$$H_{cat} = \frac{1}{N}\sum_{i=0}^{N} Ei \quad \text{---(12)}$$

Where:

Z_{cat} = The threshold used to define CHE

H_{cat} =The proportion of households (headcounts) with a catastrophic payment

N = Total sample size

E_i = Status of CHE

Furthermore, the households were sorted by per-capital household expenditure and divided into five quintiles in order to calculate the incidence of catastrophic medical payment by level of household expenditure.

E. Measuring the intensity of catastrophic payments

According to Wagstaff (2008) [5], the following equation was employed to predict household overshoot.

$\mathrm{Oi} = Ei\left(\left(\frac{\mathrm{Ti}}{\mathrm{Xi}}\right) - z\right)$ ------------------------------------(13)

Where O_i is household overshoot catastrophic payment. And the average catastrophic payment overshoot (G_{cat}) was calculated as follows:

$G_{cat} = \frac{1}{N}\sum_{i=0}^{N} Oi$ ---*(14)*

The distribution of catastrophic payments is critical in determining the disparity in consequences between the rich and the poor. The distribution of catastrophic payments among household expenditures is not shown in both incidence and intensity statistics. Better-off/worse-off concentration indices range from -1 to 1. It was possible to detect an overshot incidence (C^E) and intensity (C^O) concentration between the rich and the poor [5]. For example, when CE is negative, the risk of catastrophic payment is high among the impoverished (worse-off are most likely to exceed the threshold). If C^O is positive, it means that overshoot is concentrated in the hands of the wealthy. The method for calculating C^O and C^E in detail can be found in paper of Xu (2008) [5].

The impact of OOP payment on headcount and overshoot may be influenced by differences in household expenditure levels (high vs low) for a variety of reasons. As a result, it is preferable to reweight the spending level by assigning a high weight (2) to low expenditures and decreasing the

weight as household expenditure increases (0) [5,6]. The mean positive gap (MPG) was also determined. The MPG compares all households' average expenditures above the cut-off thresholds [7]. It has estimated as:

$H^{w} = H_{cat}\ (1-C^{E})$ --(15)

$O^{W} = Oi\ (1\text{-}C^{O})$ --(16)

$MPG = \frac{O^{w}}{H_{cat}}$ --(17)

Where:

C^{E} = The overshot concentration of incidence

C^{O} = Concentration indices of overshoot

H_{cat}= Catastrophic payment headcount

H^{w} = Weighted headcount

MPG= Mean Positive Gap

O^{W} = Weighted Overshoot

F. Measuring poverty and impoverishment

Poverty headcount

To begin, the gross medicine OOP payment poverty ratio (HP^{gross}) was calculated to determine the prevalence of people living in poverty.

$HP^{gross} = 1/N\ \Sigma\ 1\ (Xi \leq PL)$ -- (18)

Where HP^{gross} is the gross medicine OOP payment poverty headcount ratio. The function uses a bivariate variable, therefore if the personal expenditure is below the poverty line, it returns “1.” Otherwise, “0” will be used. Then, to determine the poverty headcount inconsiderate medicines OOP payment, the net of medicine OOP payment was determined. As a result, it is a percentage of poor people prior to payment of medicine. Therefore,

$HP^{net} = 1/N \Sigma 1 ((Xi-T_P) \leq PL)$ --(19)

Where:

HP^{net} = Net of medicine OOP payment poverty headcount ratio.

T_P = Per-capita OOP medicine payment

Finally, the burden of healthcare OOP expenditure was estimated by the difference between equations (18) and (19) as follows:

The burden of medicine OOP payment = $HP^{net} - HP^{gross}$ --------------------------(20)

Poverty gap

The gross and net poverty gaps have been calculated.

$G^{gross} = 1/N\Sigma 1 (Xi- PL) \mid if\ i= poor$ ---(21)

$G^{net}= 1/n \Sigma 1 ((Xi-T_P)- PL) \mid if\ i= poor\ after\ netting\ OOP$ ------------------------------ (22)

Where:

i= Household

G^{gross} = Gross poverty gap

G^{net} = Net poverty gap

References

1. WFB. Ethiopian people [Internet]. 2020 [cited 2020 Nov 30]. Available from: https://teodora.com/wfbcurrent/ethiopia/ethiopia_people.html

2. Central statistical agency. Central statistical agency survey results for: Country level. 2018;

3. Central statistical agency. The federal democratic republic of central statistical agency the 2010 / 11 Ethiopian households consumption – expenditure (HCE) survey results for country level. 2012.

4. Central statistical agency. The federal democratic republic of Ethiopia national development and planning commission central statistical agency: Country and regional level consumer price indices (CPI). 2016.

5. Wagstaff A LMOO van DE, Xu K. Analysing health equity using household survey data: a guide to techniques and their implementation. Bull World Health Organ [Internet]. Washington, DC: World Bank Publications; 2008;86:816–816. Available from: https://books.google.com.et/books?id=8krsjfKv2vgC

6. Wagstaff A, van Doorslaer E. Catastrophe and impoverishment in paying for health care: With applications to Vietnam 1993-1998. Health Econ. 2003;12:921–34.

7. David B. and Evans GC. Distribution of health payments and catastrophic expenditures Methodology [Internet]. FER/EIP Discuss. Pap. ; 2005. Available from: http://whqlibdoc.who.int/hq/2005/EIP_HSF_DP_05.2.pdf
