# Supplementary material for: The financial burden of out of pocket payments on medicines among households in Ethiopia: analysis of trends and contributing factors
Source: BMC Public Health. 2023 May 3;23:808. doi: 10.1186/s12889-023-15751-3 (PMC10155387; doi:10.1186/s12889-023-15751-3)
Supplement: Supplementary file 2 — Additional file 2. [file 12889_2023_15751_MOESM2_ESM.pdf]

Legends of Tables

Table-A 1:Item code of health service expenditure household consumption expenditure questioner

Heads of expenditure	Item code in HCE questionnaire
Healthcare expenditure on public health facilities	501
Healthcare expenditure on private health facilities	502
Other healthcare expenditures	503
Cost of transport and communication tools	601

Data source: *Ethiopian Central Statistical Agency, Household Consumption Expenditure Surveys 2010/2011 and 2015/16; available in* ***http://www.csa.gov.et***

Table-A 2: Health consumer price index (CPI) value of 2010/11 and 2015/6

Year	CPI	Used in the formula
2010/11	204.4	Old CPI
2015/16	145.9	
2021	198.1	New CPI

Data source: ***http://www.csa.gov.et***

Table-A 3: Monthly per-capita expenditure on medicine (in current price) among households in Ethiopia, 2010/11and 2015/16.

**Quintile**	**Monthly per capita consumption expenditure (OOP)**											
	**2010/11**						**2015/16**					
	Health			Medicine			Health			Medicine		
	%	µ (ETB)	P	%	µ (ETB)	P	%	µ (ETB)	P	%	µ (ETB)	P
Poorest	11	18.1	0.000*	10	6.6	0.001*	13	32.5	0.000*	11	7.7	0.000*
Poorer	16	24.2		16	10.0		16	41.2		14	9.8	
Middle	17	28.2		17	10.8		18	44.4		17	11.9	
Richer	21	35.0		20	13.0		23	56.8		24	16.7	
Richest	35	56.9		37	23.5		30	75.6		34	23.7	

%: proportion of quintile from consumption expenditure; µ: the average monthly OOP health services per-capita expenditure by quantile
*Ordered logistic regression; *p<0.05, Log likelihood = -25489.054; -29806.691, LR chi2 = 388.71;199.77; OOP: Out of-Pocket-Payment*

Table-A 4: Impoverishment impact of out-of-pocket payment (OOP) attributed to medicine expense among Ethiopian households in 2010/11 & 2015/16. (At international poverty line)

		Gross health payment (1) **		Net of health payment (2) ***		Difference			
						Absolute (3) = (2)-(1)		Relative [(3)/ (1)] *100	
**Year**		**2010/11**	**2015/16**	**2010/11**	**2015/16**	**2010/11**	**2015/16**	**2010/11**	**2015/16**
Poverty line (USD/Day)		1.90	1.90	1.90	1.90	1.90	1.90	1.90	1.90
Poverty headcount	Health	13%	4.14%	1%	4.41%	0.7%	0.27%	10.6%	7%
	Medicine			0.76%	4.18%	0.13%	0.04%	20.6%	0.97%
Poverty Gap (ETB current price[a])	Health	45.2	108.6	57.7	120	12.6	11	28%	25%
	Medicine			47.4	121.8	2.3	13.2	5%	12%
Normalized poverty gaps	Health	4.7%	8.6%	6.0%	9.5%	1.3%	0.9%	28%	0.1%
	Medicine			5%	9.62%	0.2%	1.0%	5.1%	12%

The exchange rate of USD to local currency unit (LCU) in 2011, 2016 and march of 2021was 17.18, 22.2, 40.8 and respectively.
An international poverty line of 1.90$ PPP was used to calculate the impoverishment
** PPP: Purchasing Power Parity; ETB: Ethiopian Birr, USD: United States Dollar*
[a] current price was calculated using the CPI ratio converting method
***The poverty headcount and gap estimate before netting out the health & medicine expenditure*
****The poverty headcount and gap estimate after netting out the health & medicine expenditure*

Table-A 5: Bivariate analysis of catastrophic medicine expenditure concerning household characteristics in Ethiopia, 2010/2011 & 2015/16

Probability of Catastrophic medicine expenditure									
Household characteristics		2010/11				2015/16			
		Estimated population(N)	***%***	***LR chi^2***	***P***	***Estimated population(N)***	***%***	***LR chi^2***	***P***
Sex of head	Male	300,748	0.69	3.31	0.077	159,427	0.29	25.73	0.000
	Female	98,426	0.23			242,092	0.43		
Residence	Rural	267,939	0.61	43.15	0.000	79,713	0.14	11.93	0.001
	Urban	131,235	0.3			321,806	0.58		
Household size	1	76,554	0.18	3.98	0.047	8,857	0.02	10.38	0.001
	2	62,884	0.14			38,380	0.07		
	3	71,086	0.16			67,904	0.12		
	4	106,629	0.24			82,666	0.15		
	5+	82,022	0.19			203,712	0.37		
HH Age	Adult<55	289,811	0.66	3.54	0.056	321,806	0.58	3.11	0.066
	Adult>55	109,363	0.25			79,713	0.14		
HH Education	None	87,490	0.20	2.48	0.116	162,379	0.29	29.02	0.000
	Primary	218,725	0.50			121,046	0.22		
	Secondary	79,288	0.18			29,523	0.05		
	Post-secondary	13,670	0.03			88,570	0.16		

HH Employment	Employed	0	0.00	0.00	0.000	336,567	0.60	0.022	0.641
	Unemployed	399,174	0.91			64,952	0.12		
Type of health service	In-patient service	-	-	*NA*	*NA*	259,806	0.47	13.78	0.000
	Out-patient service	-	-			144,665	0.26		
Quintile	1	144,906	0.33	47.36	0.000	239,140	0.43	141.62	0.000
	2	114,831	0.26			94,475	0.17		
	3	79,288	0.18			44,285	0.08		
	4	30,075	0.07			17,714	0.03		
	5	30,075	0.07			5,905	0.01		
Health Insurance	Insured	8,202	0.02	-0.000	0.000	2,952	0.01	2.47	0.038
	Uninsured	390,972	0.90			398,567	0.72		

LR chi^2: Logistic Regression chi-square; NA: Not Available
